# Clinical data comparison for FDA-approved gene therapies in sickle cell disease

**DOI:** 10.3389/ebm.2025.10806

**Published:** 2025-12-08

**Authors:** Alexis Leonard, Julie Kanter

**Affiliations:** 1 Department of Hematology, St. Jude Children’s Research Hospital, Memphis, TN, United States; 2 Division of Hematology, University of Alabama at Birmingham, Birmingham, AL, United States

**Keywords:** autologous transplantation, gene therapy, exal-cel, lovo-cel, sickle cell disease

## Abstract

Sickle cell disease (SCD) is a severe inherited hemoglobinopathy with limited curative treatment options. In December 2023, the U.S. FDA approved two autologous gene therapies, lovo-cel (bluebird bio) and exa-cel (Vertex/CRISPR Therapeutics), offering potentially transformative outcomes. We performed a comparative analysis of these therapies based on published clinical trial design, patient eligibility, manufacturing requirements, and reported efficacy and safety outcomes. Overall, participants treated with lovo-cel had more severe baseline disease, reflected by a higher median rate of vaso-occlusive events (VOEs), despite the use of a more stringent VOE definition. Mobilization of hematopoietic stem cells (HSCs) with single-agent plerixafor proved challenging in both trials, with most participants requiring multiple mobilization and apheresis cycles. A greater proportion of exa-cel participants required three or more apheresis procedures, driven by higher CD34^+^ cell dose targets needed to compensate for CRISPR-associated HSC loss. Both therapies demonstrated greater than 90% resolution of severe VOEs, with near-complete resolution in pediatric participants. A small subset of participants experienced VOEs post-treatment, including events occurring beyond the primary efficacy assessment period. Notably, no recurrent strokes were reported among lovo-cel treated participants with a history of overt stroke. Both therapies provide durable, clinically meaningful benefit and represent a major advancement in SCD management. However, differences in trial populations, cell collection logistics, and manufacturing have important implications for real-world applications. Continued long-term follow-up and the establishment of standardized post-treatment registries will be critical to fully assess durability, monitor late effects, and inform patient selection.

## Impact statement

This work provides the first detailed, side-by-side clinical comparison of the two FDA-approved autologous gene therapies for SCD, lovo-cel and exa-cel, integrating updated efficacy and safety data following regulatory approval. By analyzing key differences in trial design, patient eligibility, manufacturing logistics, and clinical outcomes, this study offers critical insight into how these factors influence treatment implementation and patient selection in real-world settings. The inclusion of maturing post-approval data advances the field by highlighting the durability of therapeutic benefit and emerging patterns of response across diverse patient subgroups. This comparative framework fills a knowledge gap for clinicians, policymakers, and treatment centers navigating gene therapy adoption, and underscores the need for long-term follow-up and standardized outcome tracking. Ultimately, the findings help shape a more nuanced understanding of how transformative therapies can be safely and effectively integrated into comprehensive SCD care.

## Introduction

Sickle cell disease (SCD) is an inherited monogenic disorder caused by homozygous inheritance of a single nucleotide mutation in the β-globin gene (HbSS) or by co-inheritance of HbS with another unstable β-globin variant. This results in chronic hemolytic anemia, recurrent vaso-occlusive events (VOEs), infectious complications, and reduced life expectancy [1]. Current disease-modifying therapies (DMT) with the strongest evidence for benefit include hydroxyurea (HU) and chronic blood transfusions. Both can alleviate symptoms but are lifelong, require close monitoring, are not universally effective, and can be associated with complications such as iron overload and alloimmunization. Newer DMTs offer incremental benefit: L-glutamine provides modest reductions in VOEs [2], crizanlizumab did not demonstrate superiority over placebo in a phase 3 trial [3], and voxelotor improves hemoglobin but with uncertain long-term clinical benefit and was voluntarily withdrawn from the market in 2024 [4, 5]. As these therapies fail to prevent progressive organ damage, many patients and families consider risky but potentially curative options such as allogeneic hematopoietic stem cell (HSC) transplantation, yet this is limited by donor availability, the need for immunosuppression, and risks of graft rejection and graft-versus-host disease [6]. Gene therapy approaches, including the delivery of a functional gene into or genetic modification of a patient’s own HSCs, aim to overcome these limitations and offer durable disease control. Over the past decade, advances in autologous gene therapy have led to meaningful clinical progress [7]. In December 2023, the U.S. Food and Drug Administration (FDA) approved two autologous HSC gene therapies for individuals with SCD aged 12 years and older who have a history of VOEs [8], marking a significant milestone and expanding the short list of FDA-approved therapies for individuals with SCD.

Lyfgenia (lovotibeglogene autotemcel, lovo-cel, by bluebird bio, Inc.) is a one-time *ex-vivo* lentiviral-mediated HSC gene transfer of an anti-sickling adult β globin, HbA^T87Q^. Casgevy (exagamglogene autotemcel, exa-cel, by Vertex Pharmaceuticals), is a one-time *ex-vivo* clustered regularly interspaced short palindromic repeats (CRISPR)/Cas9 based editing of the BCL11A erythroid specific enhancer in HSCs to reactivate fetal hemoglobin (HbF). Lessons learned during an early phase of the bluebird bio clinical trial were shared widely, and have resulted in an generally accepted treatment process for gene therapy delivery, regardless of product, to individuals with SCD [9]. These include specific care needs pre-transplant such as discontinuation of HU and initiation of chronic red blood cell (RBC) exchange transfusions, unique considerations during stem cell mobilization and apheresis, and targeted busulfan myeloablation. Beyond differences in the type, quantity, and hemoglobin type produced, HbA^T87Q^ in lovo-cel versus HbF in exal-cel, the two pivotal clinical trials exhibit critical distinctions that must be considered to accurately interpret their outcomes and inform clinical application post-FDA approval. Tables 1–3 provide a detailed comparative summary of these trials based on published data submitted to and included in the FDA package insert [11–14]. Updated data can be found in subsequent publications or presentations [10, 12]. Key differences encompass patient inclusion and exclusion criteria, HSC mobilization and collection protocols, as well as efficacy and safety endpoints. These aspects are herein discussed to provide a comprehensive understanding of the therapeutic profiles of each HSC gene therapy product.

**TABLE 1:** Clinical Trial Design Lovo-cel vs. Exa-cel.

	Lovo-cel	Exa-cel
Population	≥12-≤50 years (HGB-206) or ≥2-≤50 years (HGB-210) Diagnosis of SCD, with β^S^/β^S^, β^S^/β^0^, or β^S^/β^+^	12–35 years Diagnosis of SCD, with β^S^/β^S^, β^S^/β^0^, or β^S^/β^+^
Inclusion	≥4 VOEs in the 24 months prior to consent HU failure/intolerance Performance status ≥60%	≥2 severe VOCs per year in the previous 2 years Normal TCD Severe SCD with supportive care (pain plan, HU) Performance status of ≥80%
Exclusion	Available MRD History of abnormal TCD requiring ongoing transfusions Stroke^a^ Severe vasculopathy Liver, cardiac, pulmonary, renal, or infectious disease 2 or more alpha globin gene deletion^b^	Available MRD History of abnormal TCD Regular RBC transfusions that cannot be stopped Severe vasculopathy Liver, cardiac, pulmonary, renal, or infectious disease HbF >15% irrespective of HU Patients >10 unplanned hospitalizations or ER visits related to SCD consistent with chronic pain
Mechanism of action	Gene addition using lentiviral vector insertion of HbA^T87Q^	CRISPR/CAS9 editing of the erythroid-specific enhancer of *BCL11A* to reduce erythroid-specific expression of *BCL11A*
Primary outcome	Proportion of participants with complete resolution of VOEs between 6 months and 18 months after drug product infusion	Proportion of participants free of severe VOCs for ≥12 consecutive months (VF12)
VOE definition	Acute pain lasting >2 h and requiring care at a medical facility ACS: new pulmonary infiltrate with pneumonia-like symptoms, requiring O2 and/or blood transfusion Hepatic sequestration: Increase liver size with RUQ pain, abnormal LFTs, drop in Hb ≥2 g/dL Splenic sequestration: an enlarged spleen and an acute decrease in Hb of ≥2 g/dL Priapism lasting >2 h and requiring a visit to a medical facility *Severe VOE defined as VOE requiring 24h of management in hospital or observation unit OR 2 visits to ER/day unit in 2 days with both needing IV treatment*	Acute pain requiring visit to a medical facility and admin of pain meds (opioids or IV NSAIDs) or RBC transfusion ACS: new pulmonary infiltrate with pneumonia-like symptoms, pain, or fever Splenic sequestration: an enlarged spleen, LUQ pain, and an acute decrease in Hb of ≥2 g/dL Priapism lasting >2 h and requiring a visit to a medical facility *Severe VOE defined as above. Inpatient hospitalization for severe VOE was separate end point (hospitalization-free for ≥12 consecutive months HF12)*

CRISPR: clustered regularly interspaced short palindromic repeats; ER: emergency room; Hb: hemoglobin; HbF: fetal hemoglobin; HU: hydroxyurea; LFTs: liver function tests; LUQ: left upper quadrant; MRD: matched-related donor; NSAIDS: non-steroidal anti-inflammatories; O2: oxygen; RBC: red blood cell; RUQ: right upper quadrant; SCD: sickle cell disease; TCD: transcranial doppler; VF: vaso-occlusive free; VOC: vaso-occlusive crisis; VOE: vaso-occlusive event.

^a^ Patients with a history of stroke were included in early inclusion criteria, but were later excluded.

^b^ Patients with a history of two or more alpha globin deletions were included in early inclusion criteria, but were later excluded.

**TABLE 2:** Demographic and Baseline Clinical Characteristics Lovo-cel vs. Exa-cel.

	Lovo-cel	Exa-cel
Demographics		
Patients Enrolled, n	59	63
Patients Treated, n	47 as of February 13, 2023	44 as of June 14, 2023
Male sex, n (%)	28 (59.6%)	24 (55%)
b-globin genotype, n (%) β^s^/β^s^ β^s^/β^0^ β^s^/β^+^	46 (98%) 1 (2%) 0 (0%)	40 (91%) 3 (7%) 1 (2%)
Genotype for α-globin, n (%) αα/αα αα/-α3.7 -α3.7/-α3.7	32 (68.1%) 13 (27.7%) 2 (4.3%)^a^	24 (54.5%) 15 (34.1%) 2 (4.5%)
Age at enrollment in years, median (min, max) Adult, ≥18 years, n (%) Adolescent, ≥12 to <18 years, n (%)	23 (12, 38) 37 (78.7%) 10 (21.3%)	21 (12, 34) 32 (73%) 12 (27%)
Baseline clinical characteristics		
Annualized # of adjudicated VOEs, median (min, max)	3.5 (0.0, 16.5)	4.1 (2.0, 18.5)
Annualized # of adjudicated severe VOEs^b^, median (min, max)	3.0 (0.0, 13.0)	2.7 (0.5, 9.5)
Total Hb gm/dL, median (min, max) or mean +/- SD	8.7 (6.1, 12.5)	9.1 +/- 1.6
HbF (%), mean +/- SD	NA	5.4 +/- 3.9
History of stroke, n (%) Overt stroke Silent stroke	4 (8.5)^c^ 21 (52.5)^d^	NA NR
Prior HU use, n (%)	40 (85.1%)	NR

Hb: hemoglobin; HbF: fetal hemoglobin; HU: hydroxyurea; VOE: vaso-occlusive event; NA: not applicable; NR: not reported.

^a^ Patients with a history of two or more alpha globin deletions were included in early inclusion criteria, but were later excluded.

^b^ VOE, requiring inpatient hospitalization.

^c^ Patients with a history of stroke were included in early inclusion criteria, but were later excluded.

^d^ Among all treated participants across HGB-206, of HGB-210 (N = 67), 40 had MRI, data available at screening; retrospective review identified evidence of silent stroke in 21 (52.5%) [10].

**TABLE 3:** Treatment Characteristics and Outcomes Lovo-cel vs. Exa-cel.

	Lovo-cel	Exa-cel
Treatment and Engraftment Characteristics		
Number of mobilization cycles, n (min, max)	2 (1, 4)	2 (1, 6)
Cell dose: 10^6^ x CD34^+^ cells/kg, mean (range)	6.5 (3.0, 14.0)	4.0 (2.9, 14.4)
Time to neutrophil engraftment^a^, median days (min, max)	20 (12, 35)	27 (15, 40)
Time to platelet engraftment^b^, median days (min, max)	35 (19, 136)	35 (23, 126)
Drug product characteristics		
VCN, copies/diploid genome, median (min, max)	4.0 (2.3, 6.6)	NA
Percent allelic editing in bone marrow, mean (SD)	NA	86.1 (7.5%)^c^
Outcomes		
Protocol defined VOE CR, n/N (%)	30/34 (88.2%)^d^	29/30 (97%)^e^
Protocol defined severe VOE/hospitalization CR, n/N (%)	32/34 (95%)^f^	30/30 (100%)
VOE complete resolution, n/N (%) (follow-up period)	26/34 (76%) (median follow-up 35.8 months)	37/43 (86%) (0.6–45.5 months)
Severe VOE/hospitalization CR, n/N (%) (follow-up period)	29/34 (85.3%) (median follow-up 36.3 months)	40/43 (93%) (0.6–45.5 months)
Globin response, n/N (%)	33/38 (86.8%)^g^	34/34 (100%)^h^
Proportion of total Hb comprised by HbA^T87Q^ (%), median (min, max)	44.7 (27.6, 63.2)	NA
Proportion of total Hb comprised by HbF (%), mean +/- SD	NA	43.9 +/- 8.6%^i^
Total Hb gm/dL, median (min, max) or mean +/- SD	11.8 (8.4–15.0)	12.5 +/- 1.8^j^
Duration of follow-up in months, median (min, max)	35.5 (0.3, 61.0)	19 (0.8, 48.1)
Stroke, n (%) Overt Silent^l^	0 (0)^k^ 0 (0)	N/A NR

CR: complete response; Hb: hemoglobin; HbF: fetal hemoglobin; VCN: vector copy number; VOE: vaso-occlusive event; NA: not applicable.

^a^ Defined as the first day of 3 consecutive measurements of absolute neutrophil count ≥500 cells/μL on 3 different days.

^b^ Defined as the first day of 3 consecutive measurement of unsupported (no platelet transfusion in last 7 days) platelet count ≥50,000/μL on 3 different days.

^c^ Allelic editing in the bone marrow at month 6

^d^ Defined as elimination of VOE, requiring any level of medical attention between 6- and 18-month post infusion. Thirty-four patients were VOE, evaluable.

^e^ Defined as no protocol-defined severe VOCs, for at least 12 months within the first 24 months after infusion. Thirty patients were eligible for primary end point evaluation.

^f^ Defined as elimination of severe VOE, requiring hospital admission or multiple visits to an emergency department and receiving intravenous medications or priapism requiring any level of medical attention between 6- and 18-month post infusion. Thirty-four patients were VOE, evaluable.

^g^ Defined as meeting the following criteria for a continuous period of ≥6 months: weighted average HbAT87Q ≥30% of non-transfused total Hb; AND, weighted average increase in non-transfused total Hb of ≥3 g/dL vs. baseline total Hb OR, weighted average non-transfused total Hb of ≥10 g/dL.

^h^ Defined as HbF >20% with pancellular distribution for at least 3 months starting at month 6.

^i^ Proportion of total Hb comprised by HbF (%) at month 6.

^j^ Total Hb at month 6.

^k^ Patients with a history of stroke were included in early inclusion criteria, but were later excluded.

^l^ Among 40 participants across HGB-206, and HGB-210, there were no reports of recurrent overt or silent stroke in any participant with a history of silent stroke (follow-up range, 0.9–77.0 months). Among all treated participants, 41 had MRIs, available 12 or 24 months post infusion; no new overt or silent strokes were observed [10].

## Materials and methods

We performed a comparative analysis of two genetic therapies using published articles that included information on published clinical trial design, patient eligibility, manufacturing requirements, and reported efficacy and safety outcomes. Ethics approval was not required as this article used patient data from two previously completed studies [9–12].

## Results

### Clinical trial design, definitions, and demographics

Both the lovo-cel and exal-cel trials enrolled individuals with β^s^/β^s^, β^s^/β^0^, or β^s^/β^+^ genotypes who experienced at least four vaso-occlusive events (VOEs) in the 24 months prior to consent. However, the eligibility criteria for the lovo-cel protocols (NCT02140554, NCT04293185) were broader, permitting enrollment of patients with greater comorbidities, including older individuals (up to 50 years versus 35 years in exal-cel), lower performance status (ECOG 60 versus 80 for exal-cel), and initially allowing patients with a history of stroke (excluded in exal-cel) (Table 1). Additionally, lovo-cel did not exclude patients with chronic pain, a criterion applied in the exal-cel trial (NCT03745287). Exa-cel participants were required to have a HbF level ≤15.0%, regardless of concomitant treatments such as HU. There were also minor differences in the definitions of severe vaso-occlusive events (VOEs) between the trials. The lovo-cel trial defined a VOE as pain lasting more than 2 h and classified severe VOEs as those requiring hospitalization or multiple emergency room visits within a 72-h period. In contrast, the exal-cel trial defined severe VOEs as any acute pain episode necessitating medical attention along with administration of pain medication or transfusion. Despite a stricter definition of severe VOE, the lovo-cel trial reported a higher baseline median annualized number of severe VOEs, suggesting a population with more severe SCD or possibly reflecting the inclusion of patients with chronic pain (Table 2). Additionally, the median age at enrollment was higher in the lovo-cel study, which may be attributed to a greater proportion of adolescents enrolled in the exal-cel trial (Table 2). Notably, patients with the HbSC genotype were excluded in both trials.

### HSC collection and cellular infusion

Collection of sufficient HSCs following mobilization with single-agent plerixafor remains a potentially underreported challenge due to unique apheresis difficulties in patients with SCD [15]. Although both clinical protocols recommended an HSC collection target of 16.5 × 10^6^ CD34^+^ cells/kg, a higher proportion of patients in the exal-cel trial failed to collect adequate HSCs for drug product manufacturing. Specifically, 2 of 59 enrolled participants (3%) in the lovo-cel trial were withdrawn prior to infusion due to apheresis-related complications [13, 16], compared to 6 of 58 enrolled exal-cel participants (10%) who were unable to collect sufficient HSCs [17]. While the mobilization pathway used across these and other trials was identical, the exal-cel manufacturing process results in additional HSC loss relative to lovo-cel. Consequently, the FDA label for Casgevy (exal-cel) recommends a higher total HSC collection target (20 × 10^6^ CD34^+^ cells/kg) compared to Lyfgenia (lovo-cel) at 16.5 × 10^6^ CD34^+^ cells/kg, likely reflecting increased HSC toxicity associated with CRISPR/Cas9 delivery and editing. Both studies reported a median of two mobilization and apheresis cycles to obtain a sufficient drug product; however, a greater proportion of participants in the exal-cel trial required three or more cycles (14 of 44, 32%, maximum six cycles) compared to the lovo-cel trial (7 of 47, 15%, maximum four cycles) (Table 3). Cellular engraftment following gene therapy is generally prolonged compared to allogeneic transplantation in SCD, with neutrophil and platelet engraftment occurring slightly faster in the lovo-cel versus exal-cel trials. Importantly, no adverse events related to prolonged engraftment were reported in either study.

### Primary and secondary outcomes

The primary outcome definitions differed slightly between the two studies. The lovo-cel trial measured the proportion of patients without VOEs during predefined 6- and 18-month periods post-infusion, including any earlier VOE occurrences. In contrast, the exal-cel trial assessed the proportion of patients who remained VOE-free for any continuous period of at least 12 consecutive months (VF12) (Table 1). Applying these respective criteria to the primary efficacy populations (N = 34 for lovo-cel, N = 30 for exal-cel), 88% of lovo-cel participants achieved complete resolution of VOEs between months 6 and 18 post-infusion; this proportion increases to 93% when using the exal-cel VF12 definition (Table 3). Ninety seven percent of exal-cel participants achieved VF12. Secondary outcomes related to hospitalization for VOE showed that 94% of lovo-cel participants remained free from inpatient hospitalization during the 6- to 18-month post-infusion period, compared to 100% of exal-cel participants who remained hospitalization-free for at least 12 consecutive months (HF12).

Both trials reported participants who experienced VOE outside of the protocol-defined primary and secondary outcome periods (Table 3). Median follow-up was longer for lovo-cel participants at 35.5 months (range 0.3–61.0) compared to 19 months (range 0.8–48.1) for exal-cel participants. Among the eight lovo-cel participants with at least one post-treatment VOE, three did not require hospitalization; all showed greater than 50% reduction in VOE frequency relative to baseline, alongside significant decreases in hospital admissions and length of stay. With shorter follow-up, six exal-cel participants experienced at least one post-treatment VOE, half of whom did not require hospitalization. Detailed characteristics of these post-treatment VOEs remain unknown. The FDA label for Lyfgenia reported that 17 of 35 participants (49%) were prescribed opioids for sickle-related and non-sickle-related pain up to 24 months post-infusion, including some treated with buprenorphine. Vertex has not yet reported data on opioid use post-infusion in exal-cel-treated participants.

Both studies demonstrated early and sustained increases in total hemoglobin and expression of either HbA^T87Q^ (lovo-cel) or HbF (exal-cel) (Table 3). All patients discontinued RBC transfusions, maintained total hemoglobin levels at or near-normal, and exhibited improvement or normalization of hemolysis markers. Notably, both trials reported a significant reduction, but not normalization, in reticulocyte counts, the clinical implications of which remain unclear. Clinically meaningful improvements in patient-reported outcomes, including general health, physical, emotional, social, functional well-being, and pain, were observed in both trials, although direct head-to-head comparisons are not feasible.

Among lovo-cel–treated individuals with a history of overt stroke, all remained transfusion independent with no recurrent strokes observed 44–60 months post-treatment, according to the FDA package insert. Updated data with up to 70 months of follow-up reported four participants with prior overt stroke who remained stroke free and transfusion independent, with 78.6–91.6% of red blood cells expressing HbA^T87Q^ [10]. A retrospective review of lovo-cel treated individuals across the HGB-206 and HGB-210 cohorts identified silent cerebral infarcts in 21 (52.5%) of 40 participants with available MRI data at screening [10]. Notably, no recurrent overt or silent strokes were observed in any participant with a history of silent stroke over a follow-up period ranging from 0.9 to 77 months. Tables 1–3 reflect published data summarized in the FDA package inserts, with the exception of data on stroke and silent infarction in Tables 2, 3. Here we provide updated information on stroke history and outcomes, as the absence of new events after lovo-cel represents a clinically meaningful finding not otherwise reported in the literature or subsequent trials.

### Adverse events

Adverse events observed were consistent with those expected from myeloablative busulfan conditioning and underlying SCD. Patients with two or more alpha globin deletions were later excluded from receiving lovo-cel due to a reported case of myelodysplastic syndrome (MDS) in a patient with β^s^/β^s^ and α-thalassemia trait (-α^3.7^/-α^3.7^) characterized by anemia, erythroid-restricted dysplasia, and clonal abnormalities identified by karyotyping. Although the FDA prescribing information for Lyfgenia (lovo-cel) does not formally contraindicate its use, a limitation of use has been noted for patients with α-thalassemia trait or more than two α-globin deletions based on these findings.

Each study reported one post-treatment death, neither of which was attributed to the HSC gene therapy product: the death after lovo-cel resulted from significant pre-existing SCD-related cardiopulmonary disease, and the death after exa-cel was due to respiratory failure secondary to COVID-19. The FDA label for Lyfgenia (lovo-cel) carries a ‘black box’ warning for the risk of hematologic malignancy based on two participants treated with an earlier version of the product [18, 19]. That version of the therapy utilized HSCs obtained via bone marrow harvest, involved a different manufacturing process, and treated participants without contemporary supportive transplant measures [9]. This approach resulted in insufficient cell doses and persistent sickle-related stress erythropoiesis post-treatment, yielding secondary malignancy rates similar to those observed after allogeneic transplantation in patients experiencing graft failure [20, 21]. The outgrowth of a pre-existing premalignant clone may occur following hematopoietic system regeneration after conditioning, especially in the context of ongoing host stress erythropoiesis due to ineffective therapy [22]. Importantly, although Casgevy does not carry a boxed warning for hematologic malignancy, it is important to recognize that recipients of autologous stem cell transplants face a lifelong secondary malignancy risk estimated at 1–2%. Furthermore, myeloid malignancy SCD population studies show an increased relative, even if a low absolute, risk of acute myeloid leukemia/MDS and may contribute to this risk [23]. While the currently FDA-approved HSC gene therapy products demonstrate short-term clinical efficacy akin to stable engraftment following allogeneic transplantation, the long-term risk of secondary malignancy remains unknown and warrants vigilant monitoring for all HSC gene therapy recipients, not solely those treated with Lyfgenia. The FDA mandates long-term follow-up of patients for at least 15 years post-infusion, including assessments of safety and potential development of malignancies. Unfortunately, standardized methods for implementing this long-term surveillance have yet to be established, and no universal registry exists for data collection; instead, each manufacturer is responsible for maintaining their own post-treatment patient registries.

### Data following FDA approval

Post-FDA approval data as of 2024 continue to support the transformative and durable clinical benefits of both lovo-cel and exal-cel for individuals with SCD. In the lovo-cel program, 58 participants (including 14 pediatric patients) were treated under the current manufacturing process, with a median follow-up of 47.7 months (range up to >6 years) [24]. Among 38 evaluable participants, 87% achieved complete resolution of VOEs (VOE-CR) and 95% achieved complete resolution of severe VOEs (sVOE-CR) during the 6–18 months post-infusion. All pediatric participants (10/10) met both VOE-CR and sVOE-CR endpoints. In total, 11 participants experienced VOEs after therapy, of which 6 participants experienced VOEs after the assessment period. VOE-CR was more likely in individuals with <10 annualized VOEs at baseline (97%), despite similar levels of HbA^T87Q^ expression across the cohort. Importantly, patients with prior overt or silent stroke remained stroke-free and transfusion-independent for up to 70.1 months [10].

In comparison, exal-cel has been administered to 46 participants, including 12 adolescents, with a median follow-up of 33.2 months (range, 8.9–62.2 months). Among 42 evaluable participants, 93% (39/42) achieved ≥12 consecutive months free of VOEs (VF12), and 98% (41/42) achieved freedom from VOE-related hospitalizations (HF12). All pediatric participants (12/12) achieved VF12, although one adolescent experienced a severe VOE outside the VF12 assessment window. Nine total VOEs occurred after therapy, primarily in adults with pre-existing chronic pain or in association with identifiable triggers such as infection, corticosteroid use, or surgical procedures.

## Discussion

Current evidence demonstrates that both FDA approved HSC gene therapy products markedly improve clinical outcomes and significantly transform the lives of individuals with SCD; however, the long-term durability of these effects and their impact on end-organ function remain unknown. Consequently, gene therapy should be considered transformative but not curative at this time, acknowledging that a true cure for SCD is multifaceted, encompassing biological, functional, and organ-based outcomes. Biologically, these therapies increase total hemoglobin, largely comprised of anti-sickling hemoglobin, with near normalization of hemolysis. Functionally, episodes of acute pain requiring medical intervention are substantially reduced or eliminated, although the complexity of pain phenotypes, pain memory, opioid-induced hyperalgesia, and post-treatment opioid requirements remain poorly understood. Additionally, vaso-occlusive pain specific to SCD is challenging to measure and often indistinguishable from other acute pain etiologies. Quality of life is significantly improved; however, the elimination of sickling and related complications does not erase a lifetime of living with, and identifying with, a devastating chronic disease. Long-term stability or improvement of organ function is necessary for gene therapy to be considered a durable, biological cure that provides sustained, meaningful enhancements in patient function and quality of life. Despite differences in the study designs as discussed, the available data on the biological and functional outcomes of these two FDA approved HSC therapies appear similar; however, it remains unclear whether their different mechanistic effects, HbA addition vs. HbF induction, will have an equivalent or meaningful impact on the long-term end organ function and durability.

**FIGURE 1:**
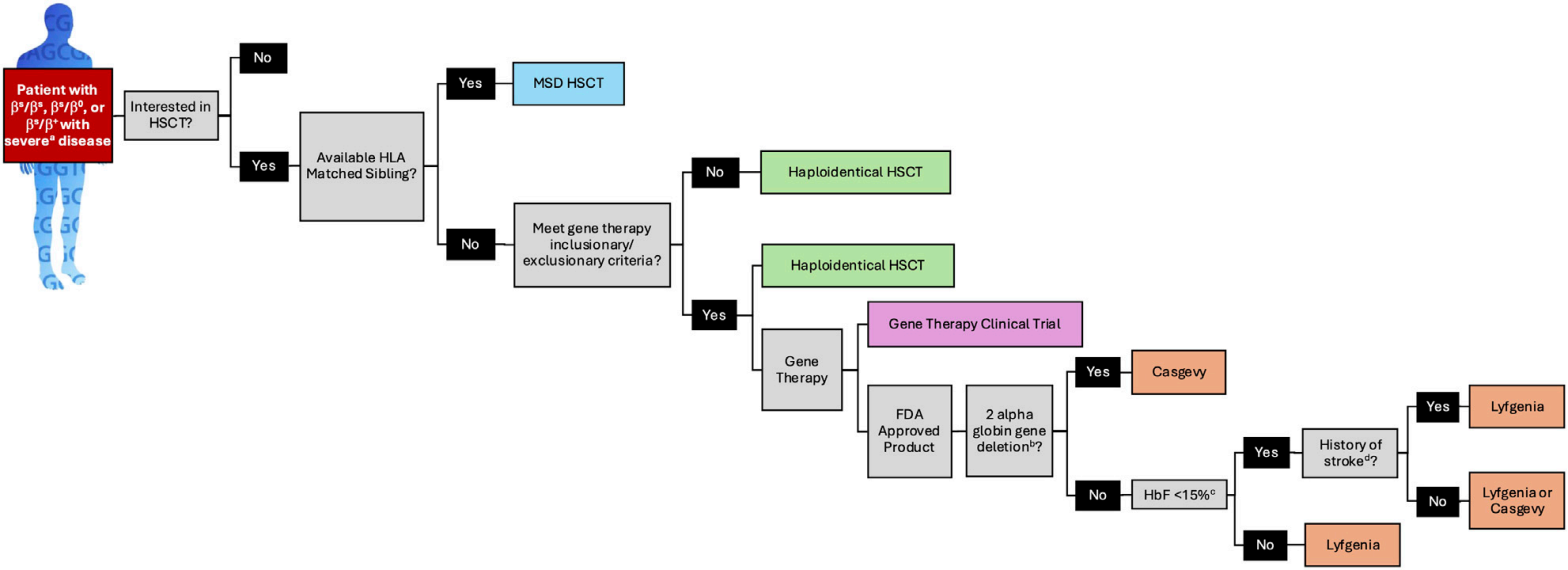
A Patient Selection Algorithm for FDA Approved SCD Genetic Therapies Based on Clinical Trial Design and Data. a. see Table 1 for definition of severe SCD as defined for lovo-cel and exa-cel. b. a limitation of use for Lyfgenia has been noted for patients with α-thalassemia trait or more than two α globin deletions. c. HbF >15% irrespective of HU was exclusionary to receive exa-cel. FDA: food and drug administration; HbF: fetal hemoglobin; HLA: human leukocyte antigen; HSCT: hematopoietic stem cell transplantation; MSD: matched sibling donor.

The lives of individuals treated on the lovo-cel and exal-cel clinical trials that led to FDA approval are profoundly transformed, offering hope to millions of individuals living with SCD. However, FDA approval represents a significant milestone rather than an endpoint in advancing the safety and efficacy of autologous HSC gene therapy for SCD. In the post-approval setting, clinical implementation of these therapies should be guided by SCD specialists, and patients should maintain access to clinical trials exploring alternative treatment approaches. While stem cell transplant physicians possess expertise in the autologous transplant process, individuals with SCD present unique clinical challenges that require specialized knowledge in SCD pathophysiology, pain management, and comprehensive psychosocial support. Cellular therapies are complex, costly, and time-intensive “one-time” treatments necessitating multidisciplinary care teams with dedicated resources tailored to SCD patients and their unique needs, including coordinated support before, during, and long after treatment. Unlike established guidelines for allogeneic transplantation [25], consensus recommendations from SCD experts regarding patient selection for gene therapy are currently lacking. In the interim, characteristics of existing clinical trials may inform patient and provider decision-making when translating trial outcomes into real-world practice (Figure 1).

The clinical use of Lyfgenia and Casgevy requires that SCD transformative therapy teams possess a thorough understanding of clinical trial data, comprehensive knowledge of alternative SCD treatments, and the ability to effectively communicate both the potential benefits and uncertainties to patients. These teams must also implement robust plans for long-term outcome monitoring and actively advocate for broader patient access to these therapies. As more sickle cell centers allocate resources to safely deliver HSC gene therapy and commit to the ongoing management of treated patients, the overall quality of care for all individuals living with SCD will improve, not merely the few who are likely to receive transformative therapy. Enhancing high-quality, comprehensive care, including the safe administration of FDA-approved therapies, remains a critical priority for all providers of and individuals living with SCD.

## Data Availability

Data is available in the manuscripts referenced given this is not primary data.
